# Intubation videoendoscopy in a preterm infant with congenital tracheal stenosis and laryngotracheoesophageal cleft: a case report

**DOI:** 10.1186/s12887-026-07339-8

**Published:** 2026-07-22

**Authors:** Yu Namikata, Kouji Motokura, Yuki Tenma, Kotaro Eriguchi, Kentaro Watanabe, Kotaro Inaguma, Shinya Okamoto, Yoshinobu Nishida

**Affiliations:** 1 Department of Pediatrics, Hyogo Prefectural Amagasaki General Medical Center, Amagasaki, Hyogo Japan; 2 Department of Pediatric Surgery, Hyogo Prefectural Amagasaki General Medical Center, Amagasaki, Hyogo Japan; 3 Department of Pediatric Cardiology, Hyogo Prefectural Amagasaki General Medical Center, 2-17-77, Higashinaniwa-cho, Amagasaki, Hyogo 660-8550 Japan

**Keywords:** Congenital tracheal stenosis, Laryngotracheoesophageal cleft, Tracheal disease, Intubation videoscope, Infant newborn

## Abstract

**Background:**

The co-occurrence of congenital laryngotracheoesophageal cleft and tracheal stenosis is extremely rare. Diagnosis is difficult and many affected neonates die within hours to days.

**Case presentation:**

A male neonate was delivered at 32 weeks and 5 days of gestation. Birth weight was 1364 g. Severe asphyxia and respiratory failure were apparent soon after birth. Endotracheal intubation was not successful and esophageal intubation did not provide effective ventilation. Flexible intubation videoendoscopy was performed immediately while continuing mask bagging, which identified congenital laryngotracheoesophageal cleft and tracheal stenosis. Considering the diagnosis, our inability to intubate, and the patient’s other anomalies including left diaphragmatic hernia and congenital heart disease, palliative care was proposed and the family agreed. He died 4 h after birth.

**Conclusions:**

Congenital laryngotracheoesophageal cleft and tracheal stenosis, a rare and mostly fatal anomaly, can be diagnosed in neonates using intubation videoendoscopy. Rapid diagnosis allows for the early introduction of palliative care.

**Supplementary Information:**

The online version contains supplementary material available at https://doi.org/10.1186/s12887-026-07339-8.

## Background

Congenital tracheal stenosis is a rare and life-threating malformation and estimated to occur in 1 in 64,500 births [1]. Cantrell and Guild classified it into three types based on the length of stenosis [2]. Up to 70% of patients with congenital tracheal stenosis have associated cardiovascular anomalies [3]. Laryngotracheoesophageal cleft is another rare congenital defect with an incidence of 1 in 10,000 to 20,000 births [4]. Such clefts are classified into four types based on severity using the Benjamin–Inglis system [5]. Between 2% and 9% of cases are associated with other congenital tracheobronchopulmonary anomalies [4].

Only 10 cases of congenital laryngotracheoesophageal cleft combined with tracheal stenosis diagnosed during the fetal or neonatal period have been previously reported. None of the reports included laryngoscopic imaging or videography [6–14]. One reason that so few cases have been reported is that many of the patients die before diagnosis. We present a neonate with both laryngotracheoesophageal cleft and congenital tracheal stenosis who was diagnosed immediately after birth by laryngeal observation using a flexible intubation videoendoscope. Rapid diagnosis allowed for the early introduction of palliative care.

### Case presentation

A 36-year-old woman (gravida 2, para 0) was referred to our hospital at 31weeks of gestation for polyhydramnios and fetal heart disease. The fetus was diagnosed with with double outlet right ventricle, pulmonary stenosis, right aortic arch, and suspected esophageal atresia. Betamethasone and magnesium sulfate were administered after rupture of membranes occurred 2 days prior to delivery. A caesarean section was performed because of severe fetal distress.

A male neonate was delivered at 32 weeks and 5 days of gestation. Birth weight was 1364 g (-2.0 SD: standard deviations), birth length was 42 cm (-0.4 SD), and head circumference was 29 cm (-0.3 SD). Apgar scores at 1 and 5 min were 1 and 2, respectively. His bradycardia resolved after 2 min of bag-mask ventilation. Endotracheal intubation was attempted five times without success and esophageal intubation did not provide effective ventilation.

After admission to the neonatal intensive care unit, his heart rate ranged between 110 and 150 beats per minute, systemic blood pressure was 53 mm Hg, and oxygen saturation ranged from 63% to 81% on bag-mask ventilation with 100% oxygen. High inspiratory pressures (approximately 30 cmH_2_O) were required to attain chest rise.

**Fig. 1:**
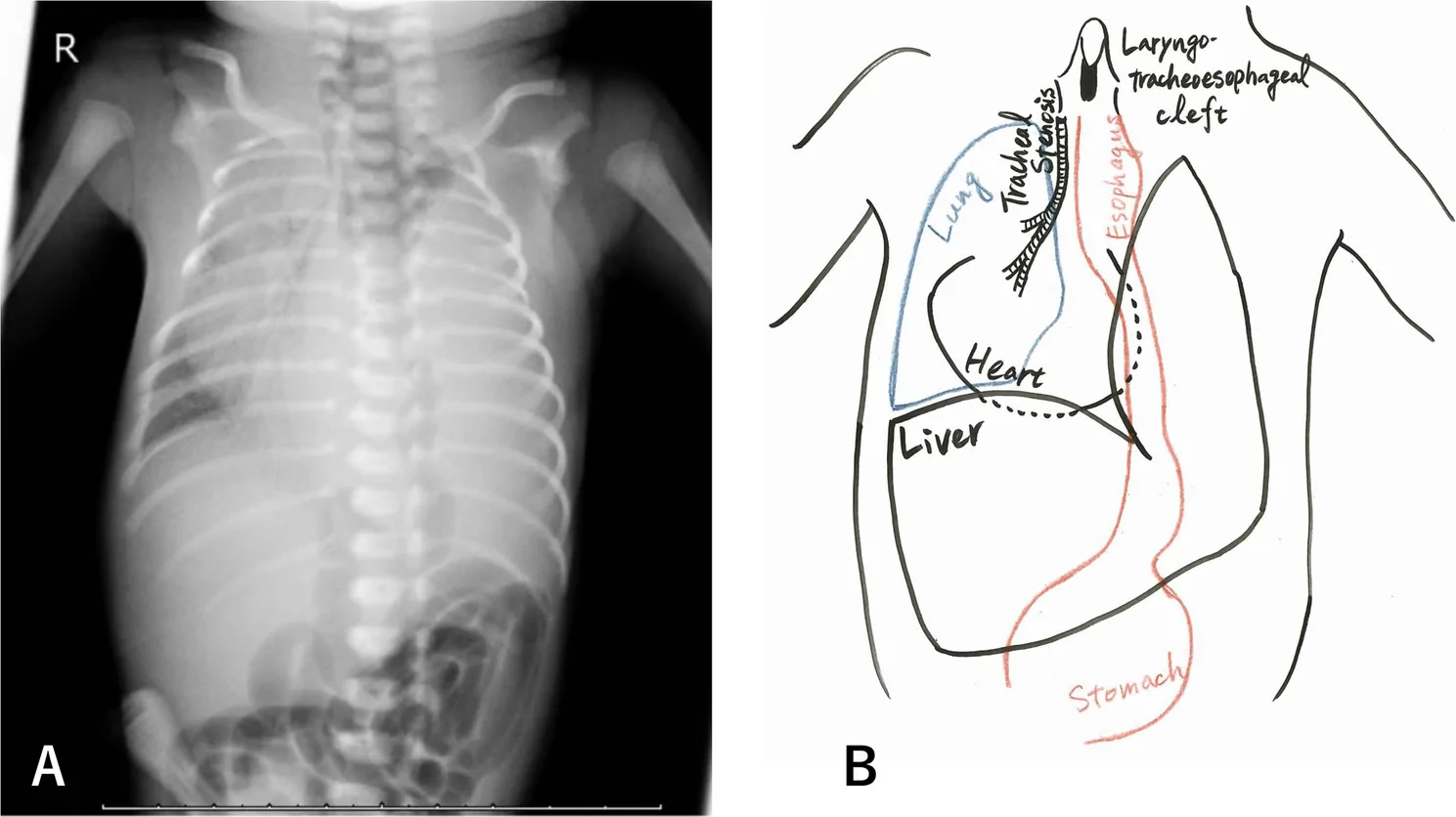
(**A**) Plain chest radiography showed poor aeration of the left lung, a narrow right bronchus, emphysema around the larynx, and deviation of the heart to the right. (**B**) A sketch showing information from fetal and postnatal ultrasonography added to the radiograph

On physical examination, he had a small jaw, but no other abnormal facial or limb features. A pediatric surgeon noted thin tracheal cartilage on palpation. Plain chest radiography demonstrated poor aeration of the left lung, a narrow right bronchus, emphysema around the larynx, and deviation of the heart to the right (Fig. 1). Venous blood pH was 6.71, pCO_2_ was 158 Torr, base excess was − 25.3 mmol/L, and lactate was 9.0 mmol/L.

To determine the cause of the failed intubation and to assess the feasibility of endoscopic intubation, laryngeal observation was performed using a flexible intubation videoendoscope (Flex.Intub.Videoendoscope 3.0 × 51.5, Set, Item no. 11301ABXK; KARL STORZ, Tuttlingen, Germany) connected to the C-MAC monitor system (KARL STORZ) while maintaining continuous mask ventilation. An endoscopy video is shown in Additional file 1. The arytenoid folds were mildly underdeveloped, no vocal cords were observed, and a laryngotracheoesophageal cleft was identified. The esophagus was located on the left. No tracheoesophageal fistula was found. A thin trachea was identified on the right ventral side with continuous air bubbles emerging. The right dorsal lumen was a blinded-end. Ultrasonography showed the liver in the left thoracic cavity and the same cardiac anomalies that were demonstrated before delivery.

These endoscopic findings explained the unsuccessful intubation attempts and indicated a complex, non-reconstructible airway anatomy. Considering the extremely poor prognosis due to multiple complex anomalies—including congenital tracheal stenosis (type 1, generalized hypoplasia), laryngotracheoesophageal cleft, left diaphragmatic hernia, congenital heart disease—as well as severe asphyxia and very low birth weight, we decided to withhold endoscopic intubation. Following a multidisciplinary discussion among neonatologists, pediatric surgeons, and cardiologists, palliative care was proposed. With the family’s agreement, comfort care was provided, and the infant died at 4.4 h of life. Postmortem computed tomography and autopsy were declined.

## Discussion and conclusions

**Fig. 2:**
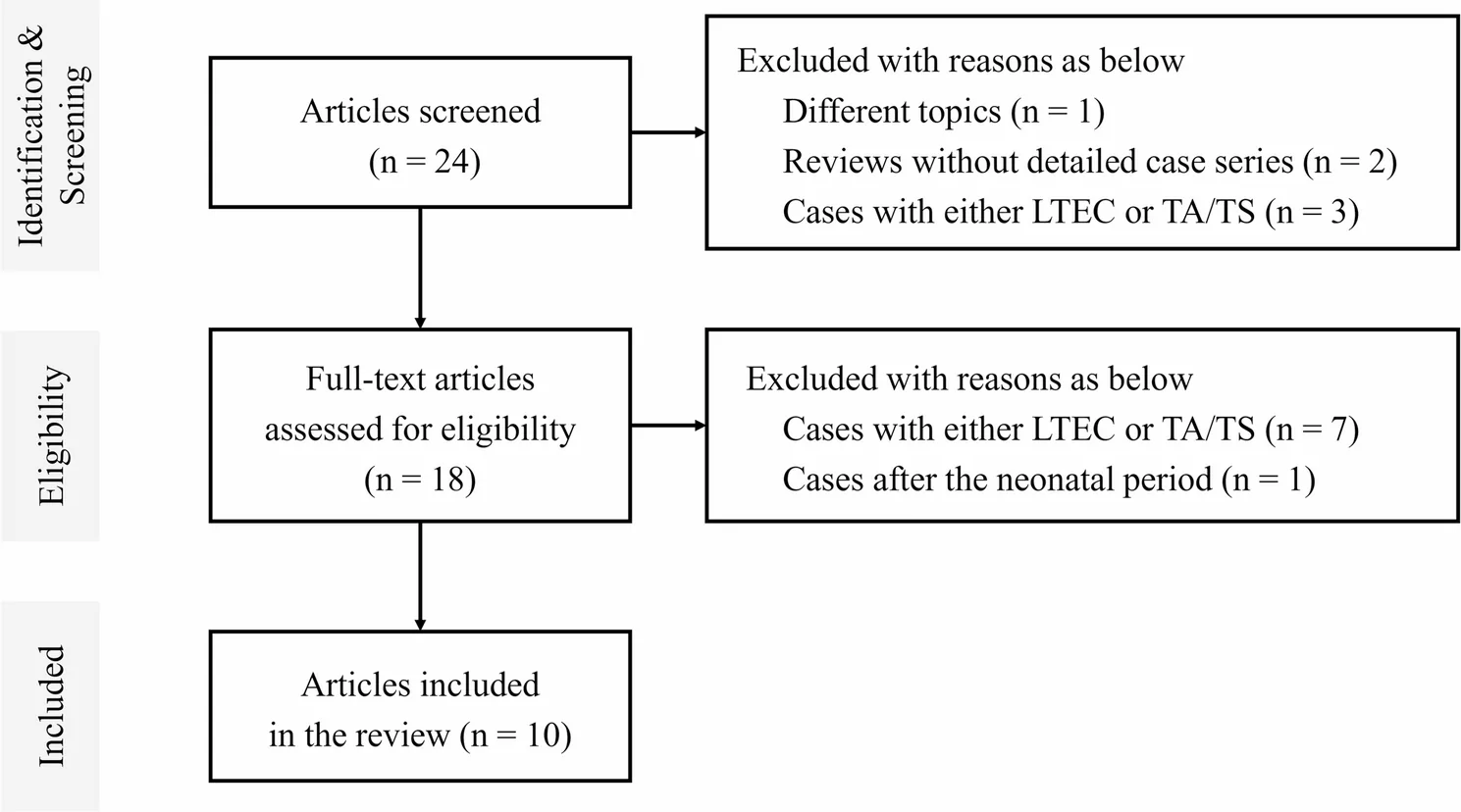
PRISMA flow chart. LTEC, laryngotracheoesophageal cleft; TA, tracheal agenesis; TS, tracheal stenosis

This is the first report to provide detailed videoendoscopic documentation of laryngotracheoesophageal cleft combined with congenital tracheal stenosis in a neonate. Co-occurrence of these congenital anomalies is extremely rare. A comprehensive search of relevant English-language studies published prior to May 2026 in the PubMed database identified only 10 cases (Table 1) [6–14]. Our search methodology and a PRISMA flow chart are outlined in Table 2; Fig. 2, respectively.

**Table 1:** Previous cases of congenital laryngotracheoesophageal cleft and tracheal stenosis/agenesis

Year Author	GA weight	Examination	Tracheal stenosis or agenesis	LTEC (type)	CHD	Outcome Other information
1976 Fuzesi	N/A 3600 g	autopsy	TA (Floyd type 3)	3 or 4	N/A	Dead (within 1 day) Rt. multicystic dysplastic kidney
1981 Novak	35 weeks 1940 g	bronchoscopy autopsy	single bronchus	4	Dextrocardia (details unk)	Dead (1 h) Multiple malformations
1983 Kingston	38 weeks 3840 g	bronchoscopy	TS	3	None	N/A Multiple malformations
1985 Ogawa	33 weeks 1765 g	bronchoscopy autopsy	right bronchial stenosis	4	DORV	Dead (8 days)
1990 Wilkinson	36 weeks 2090 g	laryngoscopy CT	TS	3b	None	Dead (11 days)
1991 Downing	35 weeks 1830 g	bronchoscopy	TS (lower segment)	small	VSD	Dead (6 months) Goldenhar syndrome
1994 Manschot	39 weeks 3050 g	bronchoscopy	TA (Floyd type 3)	dorsal 1 cm	None	Dead (10 h)
2000 Hughes	full term N/A	bronchoscopy autopsy	TS (long segment)	3b	DORV PS	Dead (3 days) Multiple malformations
2002 Lorentz	38 weeks 2170 g	laryngoscopy bronchoscopy	TS (upper segment)	1	large PDA small ASD	N/A Mos 46, XX, r(13)(p12q12)
Our case	32 weeks 1364 g	intuation videoendoscopy	TS (long segment)	2b or 3a	DORV	Dead (4 h) Multiple malformations

*ASD* Atrial septal defect, *CHD* Congenital heart disease, *CT* Computed tomography, *DORV* Double outlet of right ventricle, *GA* Gestational age, *LTEC* Laryngotracheoesophageal cleft, *PDA* Patent ductus arteriosus, *PS* Pulmonary stenosis, *Rt*. Right, *N/A* Not applicable, *TA* Tracheal agenesis, *TS* Tracheal stenosis, *unk* unknown, *VSD* Ventricular septal defect

**Table 2:** Search strategy

Database		Search Query	Results
PubMed	#1	“laryngeal cleft”[tw] OR “laryngotracheoesophageal cleft”[tw] OR “tracheoesophageal cleft”[tw]	387
	#2	“tracheal stenosis”[tw] OR “tracheal agenesis”[tw] OR “tracheal hypoplasia”[tw] OR (trachea[tw] OR tracheal[tw]) AND (stenosis[tw] OR agenesis[tw] OR hypoplasia[tw])	10,788
	#3	“infant, newborn”[tw] OR neonate[tw] OR baby[tw] OR preterm[tw] OR “premature infant” [tw] OR fetus[tw] OR fetal[tw]	1,165,542
	#4	#1 AND #2 AND #3	24

Laryngotracheoesophageal clefts were classified into four types by Benjamin and Inglis [5]. Based on videoendoscopy, the cleft in our patient was type 2 (extending below the vocal folds into the cricoid cartilage) or type 3a (extending through the cricoid cartilage but not into the trachea). His congenital tracheal stenosis was Cantrell and Guild type 1 (generalized hypoplasia). Reported mortality rates associated with type 1 tracheal stenosis range from 50% to 100% [15]. Cardiovascular anomalies occur in up to 70% of patients with congenital tracheal stenosis and are a poor prognostic factor [3]. Considering that our patient had two airway anomalies, a left diaphragmatic hernia, congenital heart disease, severe asphyxia, and very low birth weight, saving his life would have been extremely difficult, if not unlikely or impossible. Based on the best interest standard [16], we chose palliative care as the best approach for the infant to spend his limited time with his parents.

Two elements of this case warrant discussion. First, although videoendoscopy allowed us to identify our patient’s airway anomalies quite well, the final diagnosis was rendered without computed tomography or autopsy findings, which would have enabled us to fully evaluate his left bronchus and left lung. Second, to investigate whether we could have diagnosed the patient’s anomalies before birth, we reviewed the case in detail but found no evidence of increased lung echogenicity or other findings predicting congenital airway disease, except for polyhydramnios. Although fetal magnetic resonance imaging was planned, the patient underwent emergency delivery before it was scheduled.

In conclusion, congenital laryngotracheoesophageal cleft and tracheal stenosis, a rare and mostly fatal anomaly, can be diagnosed in neonates using a flexible intubation videoendoscopy. Rapid diagnosis allows for the early introduction of palliative care.

## Supplementary Information

Supplementary Material 1. Additional file 1. Flexible intubation videoendoscopy performed while continuing mask bagging showed the arytenoid folds were mildly underdeveloped, absence of vocal cords, and a laryngotracheoesophageal cleft. The esophagus was located on the left side. No tracheoesophageal fistula was found. A thin trachea was identified on the right ventral side with continuous air bubbles emerging. The right dorsal lumen was a blinded-end.

Supplementary Material 2.

## Data Availability

All data supporting the conclusions of this article, including the chest radiograph and laryngoscopy video, are included within the article and its additional file. The raw, unedited video dataset is available from the corresponding author on reasonable request.
